# Reading and White Matter Development: A Systematic Review of Neuroplastic Changes in Literacy

**DOI:** 10.3390/children12060710

**Published:** 2025-05-30

**Authors:** Yunus Pınar, Nihat Bayat, Begümhan Yüksel, Yasin Özkara

**Affiliations:** 1 Department of Early Childhood Education, Faculty of Education, Akdeniz University, Antalya 07058, Turkey; yunuspinar@akdeniz.edu.tr (Y.P.); byellice@akdeniz.edu.tr (B.Y.); 2 Department of Basic Education, Faculty of Education, Akdeniz University, Antalya 07058, Turkey; yasinozkara@akdeniz.edu.tr

**Keywords:** reading habits, reading ability, white matter, childhood brain development, diffusion tensor imaging, literacy interventions

## Abstract

**Background/Objectives:** Reading is a core cognitive ability that plays a central role in children’s brain development and academic success. This review aims to examine the neuroplastic relationship between reading acquisition and white matter development from infancy through adolescence, with a focus on implications for literacy interventions and educational planning. **Methods:** A systematic review of 89 neuroimaging studies published between 1998 and 2024 was conducted. Eligible studies utilized diffusion tensor imaging (DTI) or structural MRI to investigate white matter changes related to reading behavior in children aged 0 to 18. Studies were identified through comprehensive searches in Web of Science and Scopus databases. **Results:** Children with stronger reading abilities consistently showed increased fractional anisotropy (FA) in key white matter pathways, such as the arcuate fasciculus and superior longitudinal fasciculus, supporting phonological processing and reading fluency. Longitudinal data suggest that early exposure to literacy enhances brain flexibility and white matter organization. In contrast, children with reading disabilities, including dyslexia, often show disorganized white matter structure, but compensatory pathways may emerge through targeted interventions. **Conclusions:** Reading experience is associated with measurable changes in white matter development across childhood. Early and sustained literacy engagement appears to optimize neural structures for reading. These findings can inform early diagnosis and improve pedagogical strategies for literacy education. Further research is needed on the long-term neurobiological effects of reading interventions.

## 1. Introduction

Reading functions as a critical skill essential for both academic achievement and cognitive growth throughout childhood development, according to [1]. Modern neuroimaging research demonstrates that reading abilities and practices affect brain development through their impact on the structural integrity of white matter pathways vital for inter-regional brain communication [2]. Research using diffusion tensor imaging (DTI) has discovered important links between the characteristics of white matter and reading proficiency across populations of both children and adults. Structural variations have been linked to enhancements in cognitive functions, including attention, memory and language processing abilities which form key components of proficient reading. Reading is not only a foundational academic skill but also a powerful driver of neurodevelopment during childhood. Neuroimaging studies indicate that literacy experiences strengthen the white matter pathways that facilitate inter-regional brain communication [3,4].

Reading habits refer to the frequency and conditions of reading, while literacy encompasses the cognitive and linguistic skills required to understand written material and to acquire full literacy. In this systematic review, we use the term “reading habit” to refer to the frequency, regularity, and intensity of voluntary reading engagement, as reported or inferred in the included studies [5]. Across the studies included in this review, reading habit was assessed using a variety of methods, including self-report questionnaires, parental reports, and proxies such as print exposure frequency and the number of books available at home. In several longitudinal and interventional studies, structured reading programs and behavioral tasks were used to quantify reading engagement (e.g., 43, 78, 87). In line with the literature, we use the term “reading” as an umbrella construct that encompasses related concepts, such as reading habit, reading exposure, and reading experience, all of which refer to sustained and voluntary interaction with written language. These methodological and conceptual considerations reflect the multifaceted nature of reading behavior in the context of white matter development research. Research suggests that shared reading activities in early childhood promote phonological awareness and letter recognition skills that support later literacy development [6]. Children’s expressive language skills develop when parents participate in reading activities [7,8,9]. A study by [10] investigated the neural mechanisms behind early literacy events and how the combination of auditory and visual inputs enhances phonological processing. The arcuate fasciculus (AF), superior longitudinal fasciculus (SLF), and inferior longitudinal fasciculus (ILF) white matter pathways play a critical role in phonological processing, reading fluency, and comprehension [11,12]. Recent studies continue to explore how reading habits and skills affect white matter development. Neuroimaging studies indicate that these learning experiences can produce detectable structural changes in white matter integrity during key developmental periods [2,13].

Despite a growing number of studies on the neurobiological correlates of reading, there is a lack of synthesized knowledge regarding:

- which white matter pathways are consistently implicated across studies;
- how reading-related brain changes differ across developmental stages (e.g., preschool vs. adolescence);
- how these patterns vary between typically developing children and those with reading difficulties such as dyslexia.

This systematic review analyzes neuroimaging research outcomes to understand how reading activities impact white matter maturation in children aged 3 to 18. Specifically, we ask:

What role do reading habits and skills play in shaping white matter pathways in the developing brains of children and adolescents, and how do these effects vary across developmental stages and between typical and atypical readers?

Our research aims to develop an integrative view of structural brain changes that occur through literacy development, while examining how early experiences with reading and targeted interventions can influence brain plasticity.

### 1.1. Reading Habits

Numerous studies have examined how different reading frequencies impact cognitive development. An analysis of longitudinal data on children’s reading habits demonstrated that these habits develop primarily during their preschool years and early school years [14]. Reading frequency tends to correlate with socio-economic status (SES) because individuals with lower SES commonly show decreased reading frequency [15]. The practice of reading fosters cognitive stimulation while simultaneously improving multiple cognitive and executive functions [16]. A meta-analysis study demonstrated that children who read for leisure develop larger vocabularies compared to those who read less often [17]. Ref. [18] found that adolescents who read more often displayed superior performance in vocabulary tests as well as speech act analysis and oral narrative discourse measures. Researchers analyzed how reading habits connect to emotional vocabulary by asking 465 Swedish adolescents to generate words from emotional and neutral categories while gathering data about their reading patterns. The research showed that individuals who read often used a wider range of emotional vocabulary compared to those who read less frequently, demonstrating a relationship between reading frequency and emotional language proficiency [19].

### 1.2. Reading Skills

An individual’s executive functions and basic reading abilities, such as decoding and semantic processing, determine how well they read across various texts. The reader’s relevant background knowledge functions as a key factor that affects their comprehension of the text [20]. Reading abilities encompass the cognitive processes that enable people to translate written material into meaning through decoding and understanding. Literacy development depends on basic skills that comprise phonological awareness and vocabulary acquisition, along with reading fluency and comprehension, as identified by [21]. Phonological awareness involves recognizing linguistic sounds and manipulating them within a language system. Phonological awareness serves as an essential foundation for reading achievement because it enables learners to decode written texts [22]. Language attainment requires vocabulary acquisition because an understanding of a text depends on the reader’s ability to derive meaning from their vocabulary knowledge. Reading fluency enables learners to quickly identify words and process them efficiently. Research shows that both reading fluency, together with vocabulary acquisition, enhance reading proficiency [23]. Reading skills demonstrate a strong association with the structural integrity of brain white matter pathways, according to neuroimaging research [24,25,26]. The arcuate fasciculus serves as an essential connection between the temporal and frontal lobes that facilitates phonological processing and reading fluency [11]. The inferior longitudinal fasciculus, which connects the occipital and temporal lobes, has been linked to processes of visual word recognition and orthographic processing, according to [12]. Studies demonstrate that children who have better reading capabilities show increased fractional anisotropy (FA) values within these neural pathways, which reflects enhanced white matter integrity and connectivity. Children who struggle with reading tasks, such as dyslexic individuals [27], demonstrate diminished white matter organization in brain regions, which implies that their reading challenges stem from structural neural pathway differences.

Research into how brain structure affects reading abilities helps us to develop better literacy education methods and interventions for reading difficulties. Neuroimaging detection of white matter abnormalities early on allows for the development of specific educational approaches to improve children’s reading skills.

## 2. Methods

### 2.1. Review Framework and Search Strategy

**Figure 1:**
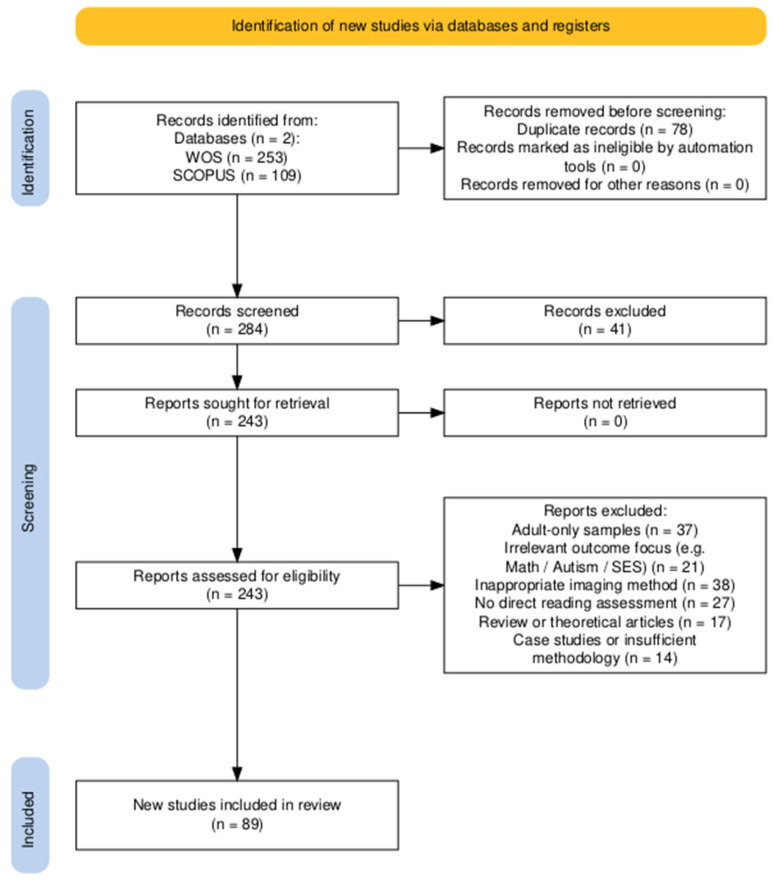
PRISMA flow diagram for study selection.

This systematic review followed the PRISMA 2020 guidelines [28]. We aimed to include neuroimaging studies examining how reading practices and skills influence white matter development in children and adolescents (aged 0–18 years). The search focused on studies published between 1998 and 2024, evaluating only those that met the predefined inclusion criteria. The first study included in the review was published in 1998, as the 1992 Shapiro study—a single case report on metachromatic leukodystrophy—did not broadly address reading development [29]. Figure 1 presents the PRISMA flowchart generated using the PRISMA2020 R package [28].

A comprehensive search was performed on the Web of Science Core Collection and Scopus databases, covering studies from 1998 to 2024. The Boolean search string, applied across both databases, was: (“reading skills” OR “reading development” OR literacy) AND (“white matter” OR “diffusion tensor imaging” OR DTI OR “structural connectivity”) AND (children OR adolescents OR pediatric).

The searches were conducted from December 2024 to March 2025, resulting in 253 records from Web of Science and 109 from Scopus. After excluding 78 duplicates, 284 studies remained for screening.

### 2.2. Inclusion and Exclusion Criteria

Our review included studies published in peer-reviewed journals between 1998 and 2024, written in English, and focused on participants aged 0 to 18 years. Eligible studies used diffusion tensor imaging (DTI) or structural MRI to explore white matter structure and function in relation to reading development, literacy abilities, or behaviors such as reading habits and fluency.

We excluded studies if they: (a) included only adult participants; (b) lacked neuroimaging techniques; (c) consisted of theoretical or review articles without empirical findings; or (d) focused on cognitive areas unrelated to reading development such as math skills or general language abilities. Studies on home literacy environments were also excluded if they did not examine links to white matter structure or connectivity.

### 2.3. Data Extraction and Analysis

We systematically extracted data from each study that met the inclusion criteria to allow for cross-study comparison and synthesis. Key variables included participant characteristics (e.g., age range, reading ability), study design (cross-sectional or longitudinal), neuroimaging methods (e.g., DTI, structural MRI), imaging metrics (e.g., fractional anisotropy, mean diffusivity), types of reading assessments or tasks used, and the main findings regarding the relationship between reading and white matter structure.

### 2.4. Study Selection Process

The researchers screened 284 studies by title and abstract, following the removal of 78 duplicate records. A total of 41 studies were excluded during this stage for not meeting the initial inclusion criteria. The remaining 243 full-text articles were assessed for eligibility. Based on the predefined inclusion and exclusion criteria, 154 studies did not qualify for inclusion in the review. The final qualitative synthesis included 89 studies that met all eligibility requirements.

### 2.5. Study Grouping

To account for methodological and developmental variation, the included studies were grouped according to study design. Cross-sectional studies investigated the relationship between white matter structure and reading abilities at a single time point. In contrast, longitudinal studies examined changes in both white matter and reading skills across different developmental stages, allowing for the observation of trajectories over time.

A standardized quality assessment tool enabled researchers to evaluate the risk of bias present in each study. The research team evaluated methodological rigor by analyzing the sample size, imaging techniques used, age range of participants, and reporting clarity. Advanced neuroimaging methods including diffusion tensor imaging (DTI) and structural MRI earned better methodological quality ratings in studies. We resolved differences in bias evaluations by holding discussions.

The PRISMA flow diagram (Figure 1) summarizes the study selection process, including the number of records screened, excluded, and included in the final analysis. The diagram was generated using the PRISMA2020 R package [28].

## 3. Results

The systematic review covered 89 neuroimaging studies, including 64 cross-sectional studies and 25 longitudinal studies. The majority of cross-sectional studies indicated a robust link between reading abilities and white matter integrity in key neural pathways, including the superior longitudinal fasciculus (SLF), the inferior longitudinal fasciculus (ILF), and the uncinate fasciculus (UF). Multiple studies consistently reported that children with reading difficulties, such as dyslexia, exhibited lower fractional anisotropy (FA) values in these regions, critical for phonological processing and reading fluency [30,31,32]. Notably, preterm children—defined as those born before 37 weeks of gestation—also showed reduced FA values in the corpus callosum, correlating significantly with poorer reading performance [33]. According to specific cross-sectional research findings, higher FA values in the arcuate fasciculus (AF) and SLF correlated with enhanced phonological awareness, fluency, and comprehension abilities [2,13,34,35,36,37,38,39,40]. Dyslexic children showed compromised white matter connectivity, particularly in the left-lateralized structures essential for phonological processing and word recognition [41,42].

Longitudinal studies further confirmed these associations, illustrating clear developmental patterns in white matter pathways related to improvements in reading proficiency. Increased reading skills were associated with higher FA values in important white matter tracts, including the arcuate fasciculus, corpus callosum, and cerebellar regions [4]. Ref. [43] notably reported differential FA trajectories, where stronger readers initially showed lower FA values that increased over time, while poorer readers had initially higher FA values that declined. Early literacy interventions further enhanced FA growth in critical pathways, like the arcuate fasciculus and inferior fronto-occipital fasciculus (IFOF) [2,44], demonstrating how reading actively shapes white matter development. Table 1 and Table 2 present systematic methodological details and key findings from cross-sectional and longitudinal studies, respectively.

Additionally, research into reading disabilities highlighted unique neurobiological differences such as atypical compensatory mechanisms in the right hemisphere of dyslexic children, characterized by increased FA in right-lateralized pathways [45,46]. These findings underline that white matter connectivity continues to adapt dynamically in response to both developmental changes and targeted educational interventions.

Research into early literacy exposure demonstrated that pre-reading children who participated in regular literacy activities exhibited enhanced white matter development, particularly in the left inferior longitudinal fasciculus (ILF) and the left uncinate fasciculus (UF), regions involved in orthographic processing and language comprehension [13,47]. Moreover, developmental dyslexic children showed reduced FA values in neural pathways, reflecting compromised white matter connectivity [46,48]. Additionally, stronger reading abilities were associated with increased fiber density in the cross-sectional area in temporo-parietal white matter regions, further highlighting structural connectivity as a significant factor in reading development [39]. A summary of the detailed characteristics, methods, and key findings of the cross-sectional studies included in this review is provided in Table 1.

**Table 1:** Cross-sectional studies.

	Authors	Study Group	N:	Measures	Results
1	Deutsch et al., 2005 [49]	Fourteen children aged 7–13 years	14	FA and CI in white matter tracts; reading, spelling, and rapid naming skills	Lower FA and CI in left temporo-parietal pathways linked to poorer reading, spelling, and naming, supporting white matter’s role.
2	Beaulieu et al., 2005 [34]	Children aged 8–12 years	32	DTI (FA in left temporo-parietal white matter); word identification (WRMT-R)	Higher FA in left temporo-parietal white matter correlates with better reading (Word ID), with the strongest correlation in the posterior internal capsule.
3	Niogi and McCandliss, 2006 [50]	Thirty-one children (6.5–10.3 years), including RD (reading disability) and non-impaired groups	31	FA in left SCR, CS, ACR; word ID, word attack, lateralization index	Higher FA in left SCR and CS correlated with better reading skills. RD group showed right lateralization, linked to poorer reading scores
4	Leonard et al., 2006 [51]	Children with DD, SLI, and mixed profiles (ages 11–16)	22	Structural MRI; anatomical risk index based on volumetric asymmetry, reading and comprehension assessments	Children with more symmetrical brain structures had severe comprehension deficits (SLI-like), while those with more leftward asymmetry had decoding impairments but preserved comprehension (DD-like). Anatomical profile predicted reading/differentiation patterns.
5	Dougherty et al., 2007 [35]	Children aged 7–12 with a range of reading abilities	49	DTI; FA and radial diffusivity in seven corpus callosum segments; phonological awareness (CTOPP), reading measures (WJ-III)	Higher phonological awareness was significantly associated with increased radial diffusivity and lower FA in the temporal-callosal segment. Suggests better readers have fewer but larger axons, indicating reduced interhemispheric connectivity may support reading.
6	Cao et al., 2008 [52]	Children with and without reading difficulties (8.9–14.11 years)	24	fMRI, Dynamic Causal Modeling (DCM), rhyming task	Children with reading difficulties showed weaker modulatory connectivity between left fusiform and parietal regions, especially on trials with conflicting phonological and orthographic cues. Reading skill was correlated with several connectivity patterns only in typical readers.
7	Odegard et al., 2009 [53]	Children with and without dyslexia (10–14 y/o)	17	DTI (FA); WIAT-II (real word), DST (pseudoword decoding)	FA in left superior corona radiata and other tracts correlated with decoding; overlap found for real and pseudoword skills. Negative correlation in corpus callosum.
8	Andrews et al., 2010 [33]	Preterm (mean age 11.9 ± 1.8 years) and term children (mean age 12.7 ± 2.5 years)	28	DTI (FA in corpus callosum and temporo-parietal region;, reading skills (WJ-III subtests)	Preterm children had lower reading scores and FA values in corpus callosum. FA in the body of corpus callosum correlated significantly with reading scores.
9	Rimrodt et al., 2010 [31]	Children with and without dyslexia, aged 7–16	31	DTI (FA, PDD); TOWRE (reading fluency), WJ-III (word ID, word attack), tractography of perisylvian network	Lower FA in LIFG and temporo-parietal WM in dyslexia; FA positively correlated with reading fluency; differences linked to fiber orientation and tract overlap.
10	Frye et al., 2011 [54]	Adolescents (~16 y), born at term and preterm	32	DTI (FA, RD, SLF volume); letter–word ID, phoneme reversal, word attack	FA and RD in left SLF significantly correlated with reading measures; SLF volume linked to reading only in preterm-born group.
11	Yeatman et al., 2011 [40]	Children aged 7–11 years	55	DTI (FA, RD in AF); phonological awareness, memory, and word reading	Higher FA in the left AF correlated with better phonological awareness and memory, with left-lateralized volume predicting reading skills.
12	Raschle et al., 2011 [55]	Pre-reading children with (FHD+) and without (FHD−) familial risk for dyslexia	20	VBM, RAN; family history, gray matter volume	Reduced GMV in left occipitotemporal and parietotemporal regions in children at risk; positive correlation between GMV and rapid automatized naming.
13	Feldman et al., 2012 [56]	Forty-two children (23 preterm, 19 full-term), aged 9–16 years	42	FA in corpus callosum, AF, SLF; verbal IQ, syntactic comprehension, decoding, and reading comprehension	Preterm group showed FA correlations with language and reading in ventral and dorsal tracts; no such links in full-term group.
14	Saygin et al., 2013 [13]	Pre-readers and early readers (Kindergarten children, aged ~5 years)	40	Diffusion MRI (FA, volume in AF, ILF, SLFp); CTOPP, WRMT-R, and RAN	Phonological awareness correlated with FA and volume in left AF; no link to RAN or letter knowledge.
15	Gullick and Booth, 2015 [37]	Children aged 8–14 years	47	DTI (FA in AF), fMRI (pSTS activity), word reading (rhyme judgment)	Better reading skills and cross-modal task performance linked to higher FA in left arcuate fasciculus, with pSTS activity predicting AF integrity.
16	Myers et al., 2014 [57]	38 children aged 5–6 years at baseline, followed until Grade 3	38	WM volume changes; reading skills	Left dorsal WM growth (such as, AF) predicted Grade 3 reading outcomes, beyond familial, env., and pre-literacy factors.
17	Horowitz-Kraus et al., 2014 [58]	Typically developing adolescents aged 15–19 years	21	DTI (FA in arcuate fasciculus and inferior longitudinal fasciculus); TOWRE-II (sight word efficiency), WJ-III (passage comprehension)	Right ILF was associated with word reading efficiency; left ILF and bilateral AF, especially right AF, were associated with reading comprehension. Findings support the Simple View of Reading, indicating distinct white matter correlates for word- and sentence-level reading.
18	Broce et al., 2015 [59]	Typically developing children (ages 5–8)	19	DWI (FA in arcuate fasciculus [segments] and frontal aslant tract); CELF-4 receptive language,	FA in bilateral AF predicted expressive and receptive language; FA in left AF increased with age; left FAT length predicted receptive language scores.
19	Skeide et al., 2015 [32]	Children aged 9–12 years	50	Phonological awareness, spelling DTI (FA of arcuate fasciculus), rs-fMRI	Genetic variant rs11100040 was associated with functional and structural connectivity (arcuate fasciculus FA) between phonological regions. Structural connectivity was linked to phonological awareness, which in turn predicted spelling and dyslexia risk scores.
20	Travis et al., 2015 [26]	Forty-five children and adolescents (9–17 years)	45	FA in cerebellar peduncles (SCP, MCP, ICP); decoding, reading comprehension	FA in MCP positively correlated with decoding and comprehension; left SCP and ICP showed negative correlations.
21	Richards et al., 2015 [30]	Grades 4–9: dysgraphia (*n* = 14), dyslexia (n = 17), controls (*n* = 9)	40	DTI (FA, RA, AD); fMRI during writing; spelling, alphabet, planning, resting state	WM integrity (FA, RA, AD) higher in controls; dyslexia and dysgraphia showed distinct WM–fMRI connectivity patterns during writing tasks.
22	Gullick et al., 2016 [38]	Typically developing children, SES stratified	42	DTI (FA in ILF, SLF, CST); real-word reading scores, SES	High SES: left ILF/SLF FA correlated with reading. Low SES: right ILF FA correlated with reading, suggesting divergent neural strategies.
23	Travis et al., 2016 [60]	Forty-two children (5.8–6.8 years), 31 Readers, 11 Pre-readers	42	FA in white matter; phonological awareness, language, and pseudoword decoding	Readers had higher FA in left aSLF and right UF; aSLF-L linked to phonological awareness, UF-R to language skills.
24	Borst et al., 2016 [61]	Typically developing children aged 9–10 years	16	Anatomical MRI (sulcal morphology of left and right OTS); oral reading (Alouette-R test)	Left OTS sulcal pattern predicted reading accuracy; interrupted OTS linked to better word reading.
25	Travis et al., 2017 [62]	Six-year-old children (readers and pre-readers)	42	Cross-sectional, FA in white matter tracts (e.g., left anterior SLF, right UF); reading skills, phonological awareness, language skills	Readers had higher FA compared to pre-readers; FA significantly correlated with phonological awareness and language skills.
26	De Moura et al., 2016 [48]	Forty children aged 8–12 years: 17 poor readers, 23 good readers	40	FA in AF, ILF, cingulum; word-level reading ability	Poor readers had lower FA in bilateral white matter tracts, indicating reduced fiber coherence.
27	Mürner-Lavanchy et al., 2018 [63]	Very preterm (VPT) and term-born children, age 7	178	DTI (FA, RD, MD), NODDI (axon density), TBSS, arcuate fasciculus tractography; language tests (semantics, grammar, phonological awareness)	Higher FA and axon density, and lower RD, AD, MD were associated with better performance in semantics, grammar, and phonological awareness in both groups.
28	Sun et al., 2017 [64]	Children with variable reading skills	66	ROBO1 genotyping, DTI (FA in corpus callosum); word-list reading.	ROBO1 polymorphisms influence reading via FA in the genu of the corpus callosum; genu FA mediates gene-to-reading effect.
29	Arrington et al., 2017 [24]	School-aged children with typical or poor decoding skills	76	Reading accuracy, fluency, comprehension, and white matter integrity (FA values)	White matter integrity in SLF, ILF, and UF correlated with reading; poor readers showed distinct tract reliance.
30	Horowitz-Kraus et al., 2017 [65]	Adolescents with mood or behavioral disorders and controls (11–17 y)	39	DTI (FA); CTOPP (phonemic awareness), WJ-III (orthographic processing, reading comprehension)	Reading skills correlated with FA in AF, ILF, SLF, IFOF; mood disorders showed lower comprehension and phonological scores and altered WM–reading associations.
31	Wang et al., 2017 [66]	Seventy-eight children (5–12 years)	78	FA, RD, and AD in AF, SLF, ILF; reading fluency, phonological awareness, and familial risk	FHD+ children had lower FA and atypical left AF lateralization; faster FA growth in right SLF aided compensation in good readers.
32	Su et al., 2018 [41]	Forty Chinese children (18 with dyslexia, 22 controls), mean age 11.1 years	40	FA in left AF (dorsal) and left ILF (ventral), phonological, morphological, and orthographic processing skills	Reduced FA in left AF linked to phonological deficits and in left ILF to morphological deficits, showing dual-pathway disruption.
33	Su et al., 2018 [67]	Chinese children (longitudinal, ages 4–14)	79	Vocabulary development (ages 4–10); DTI at age 14 (FA in arcuate fasciculus)	Children with consistently poor vocabulary growth showed significantly reduced FA in the left arcuate fasciculus, particularly in the posterior and direct segments. Vocabulary growth rate was a significant predictor of FA, independent of initial vocabulary level.
34	Lou et al., 2019 [46]	Children with developmental dyslexia (n = 26) and age-matched controls (n = 31)	57	Net. metrics (clustering, efficiency), literacy skills (reading, phonemes)	Dyslexic children had reduced left occipito-temporo-parietal connectivity, correlating with literacy skills beyond known abnormalities.
35	Banfi et al., 2019 [68]	Children with dyslexia, isolated spelling deficits, and typical peers (Grade 3)	69	DTI (FA via AFQ); SLRT-II (word and pseudoword reading), spelling test, PA, RAN, IQ	Dyslexia group showed higher FA in bilateral ILF and right SLF; FA in right ILF negatively correlated with reading; left AF FA correlated with spelling in SD group.
36	Huber et al., 2019 [69]	Children aged 7–12 with varied reading skills	53	DTI, WMTI, NODDI, Woodcock–Johnson basic reading composite	AWF and ICVF in posterior corpus callosum correlated significantly with reading skill; results robust after controlling for age and motion.
37	Dubner et al., 2019 [70]	Preterm with inflammation (PT+), preterm without inflammation (PT−), full-term (FT)	78	FA and MD in 7 corpus callosum segments; WRMT-III (reading), WASI-II (IQ), BRIEF (executive function)	Reading correlated with occipital FA (r = 0.32, *p* < 0.01); PT+ group had lower FA and higher MD in multiple callosal segments compared to PT and FT groups.
38	Broce et al., 2019 [71]	Typically developing children (5–8 y/o)	60	DTI (FA in AF, ILF, IFOF, VOF); phonological awareness; decoding	FA in AF, ILF, and VOF predicted early literacy skills; VOF newly identified as relevant for early reading development.
39	Del Tufo et al., 2019 [72]	Children with early expressive language delay	340	Reading/listening comprehension tests (e.g., QRI, WJ-PC), expressive language milestones, FA of left ILF (DTI)	Later expressive language milestones predicted poorer comprehension. Left ILF moderated the relationship. Early intervention reduced the risk of poor comprehension by 39% in at-risk children.
40	Vanderauwera et al., 2019 [73]	Adolescents aged 13–14, wide range of reading skills	34	DTI (FA, AD, RD), word and pseudoword fluency tests (Een-minuut-Test, Klepel), SES via paternal education	FA in left long AF and UF positively associated with word reading; SES also linked to FA and reading skills, suggesting environmental effects.
41	De Vos et al., 2020 [74]	Typically developing pre-readers	59	DTI (FA in left AF); auditory steady-state response, phonological awareness tasks	Rightward lateralization of 4Hz syllable-rate processing associated with higher FA in left AF; both predicted better phonological processing.
42	Beaulieu et al., 2020 [75]	Children and adolescents (aged 10–18 years)	20	myelin water fraction (MWF) imaging; standardized reading tests	Lower MWF in poor readers in corpus callosum, thalamus, and internal capsule; MWF positively correlated with reading scores.
43	Geeraert et al., 2020 [76]	Typically developing children aged 6–16 years	46	DTI, neurite orientation dispersion and NODDI, magnetization transfer imaging	White matter microstructure developed with age but showed no direct link to reading skills.
44	Hutton et al., 2020 [77]	Preschoolers aged 3–5, typical development	47	DTI (FA, AD, RD, MD); StimQ-P2 READ (HLE), EVT-2, GRTR, TRH, CTOPP-2 RAN	Higher HLE scores associated with lower AD, RD, MD in AF, ILF, UF; book reading quantity linked to higher FA and better emergent literacy skills.
45	Bruckert et al., 2020 [78]	Twenty-three children, aged 8 years (mean age = 8.2, 12 male)	23	FA and R1 in SCP, MCP, and ICP; word reading efficiency	Reading efficiency negatively correlated with FA in SCPs; no R1 link, suggesting non-myelin factors influence FA-reading associations.
46	Zhao et al., 2021 [79]	Ninety-six children aged 8–12 years,	96	FA in AF, SLF, corpus callosum, cerebellar tracts; phonological and reading fluency	Dyslexia linked to reduced left AF and SLF connectivity; better reading correlated with stronger corpus callosum and cerebellar pathways.
47	Lou et al., 2021 [80]	Children with and without reading disabilities	64	DTI, whole-brain connectome; feeder connection strength, word reading efficiency, phonemic decoding	Feeder connections between hubs and non-hubs significantly correlated with word reading efficiency and phonemic decoding; effects stronger in girls.
48	Van Der Auwera et al., 2021 [81]	Children with and without dyslexia; three time points (pre-, early-, and advanced-reading stages)	52	DTI; word and pseudoword reading (Grades 2–5), phonological awareness	FA in left arcuate fasciculus was lower in pre-readers who developed dyslexia and predicted later word and pseudoword reading skills.
49	Zuk et al., 2021 [82]	At-risk children followed from Kindergarten to Grade 2	74	DTI (FA in posterior right SLF); TOWRE-2, WRMT-III, SES, phonological awareness, speech accuracy	Higher FA in right posterior SLF predicted better decoding in at-risk children. SES, speech accuracy, and PA also predicted successful reading outcomes.
50	Wang et al., 2021 [83]	Typically developing children (n = 22) and children with reading difficulties (n = 24), aged ~9 years	46	Diffusion spectrum imaging; reading comprehension test, Chinese character recognition test	RD group showed reduced white matter integrity (GFA, NQA); reading comprehension and character recognition linked to corpus callosum indices.
51	Liu et al., 2021 [79]	Children aged 9–14 with and without dyslexia	57	DTI-based graph analysis (FA in right fusiform gyrus); word and pseudoword reading accuracy, spelling	In children with dyslexia, higher FA in the right fusiform gyrus was negatively correlated with reading accuracy, suggesting maladaptive compensation.
52	Koirala et al., 2021 [84]	Children aged 6–16 (typical and struggling readers)	412	DWI (NODDI: ODI, NDI); single word reading, phonological processing	Lower ODI and NDI associated with better reading and phonological processing; phonological processing mediated the WM–reading relationship.
53	Yu et al., 2022 [85]	Adolescents with PAE (FAS/PFAS, HE), controls	74	DTI (ILF lateralization, FA), fMRI; phonological processing, reading	FAS/PFAS group showed rightward ILF lateralization and increased right hemisphere activation; HE group showed weaker left ILF–reading correlations.
54	Gao et al., 2022 [36]	Chinese–English bilingual children, aged 8.2–12	40	DTI (FA in left/right arcuate fasciculus); English and Chinese reading tests, phonological awareness, visual–spatial ability	English reading was associated with FA in left AF (especially caudal nodes, correlated with phonological awareness); Chinese reading was associated with FA in right AF (correlated with visual-spatial ability). Findings support both language-universal and language-specific white matter mechanisms.
55	Liu et al., 2022 [27]	Fifty-seven children (9–14 years), including 26 with dyslexia and 31 matched controls	57	FA values in right fusiform gyrus (FFG); reading accuracy, pseudoword reading, spelling accuracy	Higher FA in the right FFG negatively correlated with word and pseudoword reading accuracy in dyslexic children, suggesting maladaptive compensation.
56	Meisler and Gabrieli, 2022 [39]	Children and adolescents (6–18 years)	983	Diffusion MRI (FDC, FD, FC metrics); word-reading efficiency test (TOWRE)	Higher FDC in left temporo-parietal white matter correlated with better reading; no differences between reading-disabled and typical groups.
57	Meisler and Gabrieli, 2022 [86]	Six hundred and eighty-six children aged 5–18 years, with and without reading disabilities	686	FA values in white matter tracts; TOWRE composite scores (SWE and PDE)	No significant FA differences in groups. Positive FA associations with nonword reading in older children (9+), particularly in right SLF and left ICP.
58	Brignoni-Pérez et al., 2021 [45]	Children born full-term (FT) and preterm (PT), average age: 8	79	Oral reading index, FA from dMRI, R1 metric from qT1 relaxometry	FT: Reading correlated with FA in dorsal tracts; PT: reading correlated with R1 in dorsal and ventral tracts.
59	Ostertag et al., 2023 [87]	Children with and without prenatal alcohol exposure, age 5	57	DTI (FA, AD in arcuate fasciculus); NEPSY-II (speeded naming, phonological processing), vocabulary	In PAE children, greater FA in right AF predicted better speeded naming; higher AD in left AF was linked to better phonological processing. No such associations were found in controls. Indicates altered white matter–language function relationships in PAE.
60	Harriott et al., 2023 [88]	Children with NF1 (M = 12.5 years)	28	Word reading, phonological awareness, visuospatial skills; MRI (T2/FLAIR UBO volume);	Total UBO volume significantly predicted word reading and phonological awareness, even when controlling for age, sex, scanner, and PIQ.
61	Zhao et al., 2023 [42]	Children with developmental dyslexia and age-matched controls	57	high-angular diffusion imaging (HARDI), spherical deconvolution tractography, HMOA in AF segments; reading accuracy (words, nonwords, meaningless text)	Lateralization index (LI) of AFAS correlated with nonword and meaningless text reading; LI of AFLS correlated with word reading. Findings suggest segment-specific compensatory lateralization in dyslexia.
62	Vandecruys et al., 2024 [89]	Typically developing preschoolers (mean age ≈ 5.6 years)	56	DWI; phonological awareness, letter knowledge; tracts: bilateral IFOF, ILF, anterior and direct arcuate fasciculus	Bilateral IFOF microstructure was associated with both reading and math precursors; associations were shared, not specific to reading alone.
63	Ghasoub et al., 2024 [90]	Typically developing children (ages 2–6)	81	Diffusion MRI; graph-theory analysis, NEPSY-II phonological processing, and speeded naming	Phonological processing scores positively associated with efficiency and clustering in reading-language structural networks, especially right hemisphere.
64	Cross et al., 2023 [91]	Sixty-five children aged 8–14 years	65	DTI (FA in AF, ILF, IFOF, uncinate fasciculus); single word reading, decoding, comprehension, rapid naming tasks	Higher FA in left AF associated with better decoding efficiency; higher FA in left IFOF positively linked with reading comprehension; greater FA in right ILF and bilateral uncinate fasciculus negatively correlated with reading comprehension and rapid naming skills

Notes: FA: Fractional Anisotropy; CI: Coherence Index; SCR: Superior Corona Radiata; CS: Centrum Semiovale; ACR: Anterior Corona Radiata; AD: Axial Diffusivity; RD: Reading Disability; AF: Arcuate Fasciculus; SLF: Superior Longitudinal Fasciculus; SLFp: Superior Longitudinal Fasciculus, posterior segment; ILF: Inferior Longitudinal Fasciculus; pSTS: posterior Superior Temporal Sulcus; SCP: Superior Cerebellar Peduncle; MCP: Middle Cerebellar Peduncle; ICP: Inferior Cerebellar Peduncle; OTS: Occipitotemporal Sulcus; AF-L: Left Arcuate Fasciculus; SLF-L: Left Superior Longitudinal Fasciculus; UF-R: Right Uncinate Fasciculus; MD: Mean Diffusivity; IFOF: Inferior Fronto-Occipital Fasciculus; MWF: Myelin Water Fraction; FDC: Fiber Density and Cross-Section; GFA: Generalized Fractional Anisotropy; NQA: Neurite Quantification Algorithm; WRMT-R: Woodcock Reading Mastery Tests—Revised; WJ-III: Woodcock–Johnson Tests of Cognitive Abilities—Third Edition; CTOPP: Comprehensive Test of Phonological Processing; WRMT-R: Woodcock Reading Mastery Tests—Revised; RAN: Rapid Automatized Naming; TOWRE: Test of Word Reading Efficiency; SWE: Sight Word Efficiency; PDE: Phonemic Decoding Efficiency; WRAT-3: Wide Range Achievement Test—Third Edition; WJ-III Word ID: Woodcock–Johnson III Word Identification.

Cross-sectional DTI studies have revealed that white matter integrity in the SLF, ILF, and UF may be correlated with reading; poor readers showed distinct tract dependence [24,55,66]. Studies have revealed that preterm infants have lower reading scores and lower FA values in the corpus callosum [33]. Lower FA has also been reported in the left inferior frontal gyrus (LIFG) and temporo-parietal white matter in individuals with dyslexia [13,30,55]. Some evidence indicates segment-specific compensatory lateralization in individuals with dyslexia [4]. In addition, better reading ability was associated with stronger pathways in the corpus callosum and cerebellum [92].

Longitudinal studies further support these associations, revealing that white matter integrity develops dynamically in response to reading. [43] reported that children with strong reading skills initially had lower FA that increased over time. In contrast, children with weaker reading abilities exhibited higher initial FA values that declined. This pattern suggests distinct developmental trajectories in reading-related neural pathways. In addition, early exposure to print materials and phonological instruction predicted higher FA growth in the AF and inferior fronto-occipital fasciculus (IFOF), further emphasizing the importance of early literacy interventions [16,93]. These findings highlight how early reading engagement actively influences neural plasticity and suggest that genetic and environmental factors collectively influence the development of the white matter pathways that support literacy.

Studies investigating children with reading disabilities have provided additional insights into neurobiological differences. Children with dyslexia showed atypical right-hemisphere compensation mechanisms, as evidenced by increased FA in the right fusiform gyrus and right SLF, possibly reflecting inefficient neural adaptations [45,94]. The persistence of these alterations across developmental stages suggests that white matter connectivity is not static but continues to evolve in response to environmental stimuli such as educational interventions and reading exposure.

Table 2 presents an overview of longitudinal and other studies, including the study groups, measures, and key results.

**Table 2:** Longitudinal studies.

	Authors	Study Group	N:	Measures	Results
1	Hoeft et al., 2011 [95]	Children with and without dyslexia (6–12 y/o)	45	DTI (right SLF); fMRI during phonological task; reading assessments over 2.5 years	WM organization in right SLF predicted long-term reading gains in dyslexia; behavioral predictors alone were not sufficient.
2	Yeatman et al., 2012 [4]	55 children, aged 7–15 years (39 with at least 3 measurements)	55	FA in left AF and ILF; standardized reading scores	Above-average readers had low initial FA that increased over time; below-average readers had high initial FA that declined, reflecting differing developmental trajectories.
3	Gullick and Booth, 2015 [37]	Children aged 8–14 years	30	Diffusion MRI (FA in AF); reading assessments (real-word reading, pseudoword reading)	Higher FA in the direct segment of the AF at baseline was predictive of greater improvements in reading skills over a three-year period.
4	Kraft et al., 2016 [96]	Pre-reading children with and without family risk of DD; followed into Grade 1 or 2	53	Quantitative T1 MRI (left anterior AF); behavioral literacy precursors (phonological representations, RAN); reading/spelling tests (SLRT-II, ELFE, DERET)	Increased T1 intensity (↓ myelin) in left anterior AF in risk group. Neuroanatomical predictor model (incl. AF) predicted DD better (80%) than behavioral-only model (63%). T1 of left anterior AF was significant predictor of later DD.
5	Takeuchi et al., 2016 [2]	Healthy Japanese children	296	FA; verbal comprehension scores (WAIS-III, WISC-III)	Stronger reading habits increased FA in left AF, IFOF, PCR.
6	Vandermosten et al., 2017 [44]	Pre-readers (Kindergarten children aged ~5–6)	71	Diffusion MRI (FA of IFOF and AF); cognitive reading precursors (PA, RAN), parental reading data	Paternal reading skills mediate early childhood reading outcomes via left IFOF, SES impacts white matter structure.
7	Vanderauwera et al., 2018 [97]	61 children: pre-reading stage (5–6 years) and early reading stage (7–8 years)	61	FA in ventral pathways (IFOF, ILF, UF); phonological awareness, orthographic knowledge	Left IFOF supported orthographic knowledge at early stages of reading. No ventral pathways supported phonological processes.
8	Huber et al., 2018 [98]	Struggling grade-school readers (ages 7–12)	24	Diffusion MRI (white matter metrics); reading assessments (Woodcock–Johnson, TOWRE)	Significant improvement in reading skills and widespread white matter changes over 8-week intervention.
9	Richards et al., 2018 [99]	Dyslexia (n = 20), OWL LD (n = 6), dysgraphia (n = 10), typical readers (n = 6); Grades 4–9	42	DTI (AD, MD), fMRI clustering coefficient; word, sentence, and text-level silent reading tasks pre/post 18 computerized lessons	WM–GM correlations emerged post-instruction only: (1) AD in left superior frontal ↔ right IFG (word reading); (2) MD in left superior corona radiata ↔ left MFG (sentence); (3) MD in left anterior corona radiata ↔ right MFG (text). Reading gains observed behaviorally.
10	Bruckert et al., 2019 [100]	Seventy-one children: 34 preterm (PT) and 37 full-term (FT), aged 6–8 years	71	FA in AF, SLF, ICP; phonological awareness, language, reading fluency	FA in dorsal and cerebellar pathways predicted reading outcomes in FT but not in PT children, suggesting distinct neural adaptations in PT group.
11	Lebel et al., 2018 [47]	Children (9.5 ± 1.3 years)	70	FA and MD in white matter tracts; reading fluency, phonological decoding, and sight word reading	FA increased with age in non-impaired readers, absent in dysfluent readers. Faster MD decreases in dysfluent inaccurate readers suggest compensatory changes (e.g., corona radiata, uncinate).
12	Borchers et al., 2019 [101]	Typically developing children aged 6–8 years	37	Diffusion MRI (FA in left and right SLF, left Arcuate, left ICP); cognitive assessments (language, phonological awareness)	White matter properties (FA) at age 6 in specific pathways (SLF, Arcuate, ICP) predict reading outcomes at age 8, beyond pre-literacy skills and demographic factors.
13	Su et al., 2020 [16]	Seventy-nine children; language and reading assessments from aged 1–14 years;	79	FA in AF, IFOF; early family factors (SES, literacy exposure), vocabulary growth rate	Earlier literacy exposure and SES correlated with higher FA in left AF and IFOF. Vocabulary growth rate predicted FA in left AF-posterior and AF-direct.
14	Partanen et al., 2020 [102]	87 children: 46 dyslexic, 41 average readers; aged 8–12 years	87	FA and MD in white matter tracts; voxel-based analysis of gray matter, reading fluency	Changes in white matter microstructure (lower MD) in right-hemisphere predicted reading fluency improvement. Gray matter showed no significant associations
15	Phan et al., 2021 [103]	Children aged 6–10 (typical and dyslexic readers)	41	Longitudinal T1 MRI (PBVC + scaling); reading assessments (word reading, pseudoword reading)	Cortical volume in the left reading network (temporo-parietal regions) increased during early reading stages and stabilized later. Dyslexic readers showed compensatory mechanisms in the right-hemisphere (right pars opercularis and isthmus cingulate).
16	Brignoni-Pérez et al., 2021 [45]	Very preterm infants (24–31 weeks GA), randomized to maternal speech vs. control	42	DTI (FA, MD) at 36 weeks PMA and 12 months AA; language via MacArthur–Bates CDI at 12–18 months AA	Infants exposed to increased maternal speech showed enhanced white matter development and higher language scores.
17	Zhou et al., 2022 [92]	Preschool children aged 2–7 years	73	DTI (FA, MD); phonological processing raw score, rapid naming scores	Increased FA in internal capsule and IFOF correlated with phonological processing ability; mediation analysis showed age-related changes in FA supported phonological skill development.
18	Beelen et al., 2022 [104]	Children aged 8–11 (grades 2 and 5)	43	Structural MRI (left fusiform gyrus size); reading skills (word and pseudoword reading tasks)	Early reading skills (grade 2) predicted an increase in the size of the left fusiform gyrus by grade 5. However, the size of the left fusiform gyrus in grade 2 did not predict later reading skills, indicating behavior-driven brain plasticity.
19	Manning et al., 2022 [94]	Preschool children aged 3.5–4.5 years	35	Diffusion MRI (FA, RD, AD in SLF), resting-state fMRI (functional connectivity); pre-reading skills (NEPSY-II speeded naming, phonological processing)	Structural features in SLF and functional connectivity in fronto-parietal networks at 3.5 years predicted better pre-reading skills (phonological processing, speeded naming) at 4.5 years.
20	Weiss et al., 2022 [43]	Fifty-three children; subset of 20 underwent MRI at 2 years	53	Parent–child conversational turns; FA and MPF in AF, SLF; letter knowledge and phonological awareness	Parent and conversational turns at 14 months correlated with literacy skills at 5 years. AF myelination mediated the relationship between early language input and literacy.
21	Davison et al., 2023 [105]	Children aged 5–10 (PLING cohort)	77	DTI (FA); left/right AF and SLF; lateralization index, parent-reported shared reading, standardized reading and language tests (TOWRE-2, GORT-5, PPVT-4, CELF-5)	More shared reading in Kindergarten was linked to greater FA and left-lateralized SLF, which predicted better word reading fluency in Grade 2.
22	Ghasoub et al., 2024 [106]	Children aged 3–7 with and without prenatal alcohol exposure (PAE); repeated DTI scans	135	DTI (graph theory metrics in reading network), NEPSY-II phonological processing and speeded naming	Children with PAE had lower pre-reading scores and lower graph theory metrics (global efficiency, nodal degree). PAE moderated brain–behavior associations: stronger links between phonological processing and network efficiency in PAE group.
23	Lou et al., 2024 [107]	Children aged 8–14 with and without reading disabilities	64	Diffusion-weighted MRI, connectome-based graph theory analysis of left thalamus; TOWRE-PDE (phonemic decoding), RAN (rapid automatized naming), SWE (sight word efficiency), reading comprehension	Transmission cost of the left thalamus (in reading network) was positively correlated with phonemic decoding. Local efficiency and clustering coefficient were negatively correlated with RAN. Stronger pulvinar and mediodorsal thalamic nucleus connections to temporal areas were negatively associated with decoding. Results were replicated in a validation sample.
24	Vandecruys et al., 2024 [93]	Preschool children (Mage ≈ 5.5 years); schooling vs. non-schooling groups	67	Longitudinal DTI; FA and MD in AF, ILF, IFOF, CC; word reading, letter knowledge (DMT)	Behavioral reading skills improved significantly in the schooling group, but white matter changes were driven by age-related maturation, not schooling.
25	Roy et al., 2024 [108]	Children and young adults from diverse demographic backgrounds	14,249	Reading skills (e.g., TOWRE scores), white matter integrity (FA values), socio-economic factors	Dynamic relationship between reading skill gains and white matter changes over time. No evidence of static white matter properties predicting reading skills across cross-sectional datasets.

Notes: AD: Axial Diffusivity; RD: radial diffusivity; AF: Arcuate Fasciculus; IFOF: Inferior Fronto-Occipital Fasciculus; PCR: Posterior Corona Radiata; FA: Fractional Anisotropy; SLF: Superior Longitudinal Fasciculus; ICP: Inferior Cerebellar Peduncle; ILF: Inferior Longitudinal Fasciculus; UF: Uncinate Fasciculus; WAIS-III: Wechsler Adult Intelligence Scale—Third Edition; WISC-III: Wechsler Intelligence Scale for Children—Third Edition.

The systematic analysis of the 89 included studies consistently highlighted specific neurostructural patterns linked to literacy development. The left arcuate fasciculus (AF) emerged as the most-studied white matter tract, according to studies by [37,83,108]. These studies indicated that greater FA within the left AF led to better phonological awareness, as well as improved word reading and memory skills across multiple studies [16,21,35,36,37,45,80,83,107,108]. Children who achieved higher reading scores showed strong left lateralization in this brain tract. Studies involving preterm children [33,56] revealed reduced FA scores in both the corpus callosum and temporo-parietal regions, which were associated with poorer reading skills. Early disruptions in neural development may affect the maturation of white matter structures that support literacy.

Several studies have made comparisons between typically developing children and those with risk factors for reading disability (RD). [50] showed that children with RD had right-lateralized FA patterns associated with poor reading performance. [54] observed clear differences between association and projection pathways in individuals with reading difficulties. FA emerged as the most reliable white matter metric across studies, as it typically showed positive associations with reading and language skills. Different studies showed variability in additional metrics (RD, AD, CI), resulting in some inconsistencies, primarily due to sample heterogeneity and differences in cognitive measures.

Research suggests that white matter integrity in the dorsal language pathways of the left hemisphere plays a critical role in the development of the phonological and decoding skills necessary for early reading. Research from longitudinal and early intervention studies (e.g., [13]) shows that white matter structure serves as both a marker of current skills and a predictor of future reading skills.

## 4. Discussion

This review was designed to examine how the acquisition of literacy—particularly in childhood—interacts with the structural organization of the brain, with a specific focus on white matter adaptations detectable via neuroimaging. Across 89 studies, we identified consistent associations between reading development and microstructural changes in left-hemispheric tracts, most notably the arcuate fasciculus (AF), the superior longitudinal fasciculus (SLF), and the inferior longitudinal fasciculus (ILF). Importantly, these associations did not appear in isolation but were modulated by developmental context: the child’s age, pre-existing cognitive profile, and whether reading was supported through targeted instruction or emerged through autonomous engagement. Rather than representing a linear progression, white matter development in the context of literacy appears to reflect a highly plastic and experience-dependent reorganization process.

The findings of this review provide substantial evidence that reading habits and skills play a critical role in shaping white matter development. The observed associations between FA values in key white matter tracts and literacy skills suggest that regular reading engagement actively modifies neural pathways, thus supporting experience-dependent plasticity [4,13].

A crucial finding is the impact of early reading on neural structures. Children exposed to print materials from a young age demonstrate greater maturation of the left AF and ILF, which are essential for phonological processing and word recognition [13,47]. These findings support the hypothesis that environmental stimulation drives structural brain changes [2,44].

In addition to cross-sectional evidence, longitudinal studies further support these findings by demonstrating distinct white matter developmental trajectories in skilled and struggling readers. [4] found that children with strong reading abilities exhibit progressive increases in FA values, whereas children with reading difficulties experienced a decline in FA over time, suggesting that white matter plasticity is sensitive to ongoing literacy experiences. [37] further found that children with stronger FA in the direct segment of the AF demonstrated greater reading improvement over three years. These results highlight the importance of early intervention programs to support children at risk of reading impairments.

Children with developmental reading difficulties, such as dyslexia, exhibit distinct white matter alterations, particularly in the right-hemisphere tracts, suggesting compensatory mechanisms [27,45]. Increased FA in the right fusiform gyrus and right SLF may facilitate some reading functions and indicate inefficient processing strategies, reinforcing the need for targeted interventions to enhance left-lateralized reading networks. Furthermore, studies using multimodal imaging approaches have suggested that myelination deficits in these regions contribute to reading difficulties, highlighting the role of white matter integrity in reading proficiency [75,102].

Another critical insight gained from these findings is the relationship between the environmental factors and neural plasticity. Children who engage in regular reading exhibit higher fiber density and connectivity in reading-related white matter tracts (e.g., [2,13,37,39,47,98]). Similarly, ref. [2] demonstrated that frequent reading was associated with increased FA in the IFOF and AF, emphasizing the role of sustained literacy engagement in brain plasticity. This suggests that reading interventions are beneficial for improving literacy skills and have a direct impact on brain structures.

Findings from interventional studies further support the role of structured reading programs in modifying the white matter structure. Huber et al. (2018) [98] demonstrated that an eight-week reading intervention led to widespread white matter changes, particularly in motor-integration pathways such as the posterior corona radiata and inferior cerebellar peduncle. These results confirm that reading is both a cognitive skill and a driver of neurodevelopment, and that interventions tailored to enhance reading fluency can lead to measurable neural adaptations [2,37,44,81,95,96,97,102].

Furthermore, findings from studies on preterm children indicate that early disruptions in white matter development can have long-term consequences for reading ability. [33] found that preterm children exhibited lower FA values in corpus callosum tracts, correlating with poorer reading performance in later childhood. This highlights the importance of early identification and targeted reading support for at-risk populations.

The dynamic relationship between reading habits and brain structure highlights the need for early literacy interventions, particularly for at-risk children. As neural plasticity continues into adolescence, future research should explore the long-term impact of different types of reading exposure and intervention strategies that optimize white matter development. Understanding how reading shapes the brain will inform educational best practices and provide neuroscientific insights into literacy acquisition and its broader cognitive benefits. Future studies should employ longitudinal neuroimaging methods to examine how different types of reading interventions influence structural brain changes over time and explore the role of digital reading formats in shaping white matter connectivity compared to traditional print reading.

In light of the reviewed evidence, literacy emerges not merely as a cultural skill but as a biologically embedded force capable of reshaping brain architecture. The observed plasticity within white matter pathways—particularly those implicated in phonological processing and orthographic–phonological integration—underscores the sensitivity of the developing brain to literacy-related input. While considerable variability exists across age, population, and methodology, the overall trajectory points to a consistent theme: that structured reading exposure, especially when embedded in targeted interventions, can promote measurable neuroanatomical changes. This reinforces our initial aim of synthesizing a coherent framework for understanding the neurostructural correlates of literacy acquisition. Future research integrating behavioral, longitudinal, and interventional designs will be essential to refine this framework and translate it into pedagogical and clinical practice.

### Limitations and Future Directions

This review provides a comprehensive summary of current neuroimaging research on reading and white matter development in children and adolescents; however, it is important to recognize certain limitations, including the lack of linguistic and cultural diversity in the reviewed studies. The exclusive use of Web of Science and SCOPUS as databases may have resulted in the exclusion of relevant studies indexed elsewhere. In addition, the diversity of age groups, imaging protocols and literacy measures in the included studies limited the potential for direct comparisons or meta-analyses. As part of this review, we synthesized findings from 89 neuroimaging studies and identified several consistent trends as well as important limitations. Most research utilized diffusion tensor imaging (DTI) methods to examine white matter structures like the arcuate fasciculus (AF), superior longitudinal fasciculus (SLF), and inferior longitudinal fasciculus (ILF). The bulk of research targeted children between 6 to 12 years old; only a small number of studies examined early childhood or adolescent periods. The research consistently showed white matter alterations connected to literacy but displayed significant methodological diversity through varied reading assessments, imaging parameters, and sample characteristics. The scarcity of studies investigating longitudinal trajectories and intervention outcomes restricts our knowledge of developmental timing and causal relationships. Future research would benefit from more standardized methods and greater reporting of null results to reduce publication bias. Studies that include linguistic and cultural diversity may also deepen our understanding of white matter development. The results of the review highlight the need for early identification and treatment of reading difficulties at crucial stages of brain development for practical applications. To improve reading skills and potentially support white matter development, policy makers and education systems need to adopt early diagnostic methods and specific educational programs. Collaboration between teachers, brain researchers and medical professionals can help to create literacy programs that match the developmental pathways of children’s brains.

## 5. Conclusions

The review demonstrates how children’s reading practices and abilities affect their brain’s white matter pathway development. The AF, ILF, and SLF white matter tracts demonstrate increased FA levels and myelination with stronger connectivity in children who frequently read and show high literacy proficiency.

Engaging in reading activities from an early age leads to enhanced white matter plasticity, which supports how reading habits shape neural structures. Exposure to literacy-rich environments enhances white matter pathways, leading to improved phonological awareness, reading fluency, and comprehension. Children who have reading disabilities, like dyslexia, show decreased FA and abnormal connectivity patterns, which indicates that poor white matter organization may obstruct reading skill development. Targeted reading interventions and phonological training programs produce measurable changes in white matter structure, indicating that literacy instruction can effectively influence brain adaptability.

The broader implications of these findings extend significantly beyond academic achievement, influencing both educational practices and societal outcomes. The diminishing traditional reading practices among children, together with the growing consumption of digital media, brings forward worries regarding literacy development in the future. Reading promotes deep cognitive processing and sustained attention while developing language skills, which serve as foundational elements for both academic achievement and professional advancement. Educators and caregivers, alongside policymakers, should focus on early literacy interventions and structured reading programs to enhance cognitive development and neural functions.

Through neuroimaging and cognitive assessments, professionals can identify children who are at risk early enough to provide timely interventions during their critical developmental phase. Studies conducted over extended periods demonstrate how early literacy programs reduce reading problems and confirm the critical period hypothesis stating that childhood interventions yield the best results. Research should investigate how white matter development responds differently to digital and print reading formats and evaluate the enduring effectiveness of reading interventions among varying socio-economic and linguistic groups.

Reading serves as a critical neurobiological mechanism for brain development, extending well beyond academic learning. Research demonstrates that children achieve better cognitive and neural development when they experience literacy-rich environments alongside early reading exposure and dedicated interventions. In today’s digital society, children need to build consistent reading habits to succeed. Research into the brain mechanisms involved in learning to read will inform the creation of educational policies and interventions that support lifelong literacy and cognitive health.
